# Immune responses to Epstein–Barr virus: molecular interactions in the virus evasion of CD8^+^ T cell immunity

**DOI:** 10.1016/j.micinf.2009.12.001

**Published:** 2010-03

**Authors:** Martin Rowe, Jianmin Zuo

**Affiliations:** Cancer Research UK Birmingham Cancer Centre, University of Birmingham, College of Medical and Dental Sciences, Vincent Drive, Edgbaston, Birmingham B15 2TT, United Kingdom

**Keywords:** Epstein–Barr virus, Immune-evasion, Antigen presentation, Tumour virus, Herpesvirus, Immune response

## Abstract

Persistent viruses have mechanisms for modulating the host immune responses that are essential for achieving a lifelong virus–host balance while minimizing the viral pathogenicity. Here we review some of the immune-modulating mechanisms evolved by the ubiquitous but potentially oncogenic Epstein–Barr virus, with particular emphasis on the molecular mechanisms of genes interfering with HLA class I antigen presentation.

## 1 Introduction

Persistent viruses are pathogens that co-exist in balance with and for the life of their immunocompetent hosts, often without causing any symptoms. This delicate virus–host balance can be upset by perturbation of the immune responses, or through genetic or environmental factors. Indeed, persistent viruses are a major worldwide source of morbidity and mortality, responsible for diverse pathologies ranging from virus-associated cancers to life-threatening virus replicative infections in the immunocompromised. Epstein–Barr virus (EBV), which is carried by more that 90% of the adult human population worldwide, is a prime example of a persistent virus that is generally harmless, but which can cause serious diseases.

EBV is a γ_1_-herpesvirus that replicates in permissive cells in the oropharynx but persists as a latent infection in long-lived memory B lymphocytes. Primary infection with EBV transmitted by salivary transfer is usually clinically silent but can manifest as infectious mononucleosis (IM), a self-limiting lymphoproliferative disease caused by excessive but ineffective T cell responses to EBV-infected B cells [1]. As the mononucleosis resolves, effective CD8^+^ and CD4^+^ T cell responses become established and the virus:host homeostasis is achieved.

The importance of the host T cell surveillance for preventing EBV pathogenesis is well-illustrated by the increased incidence of potentially fatal lymphoproliferative lesions in patients receiving immunosuppressive therapy following organ transplants, which can be reversed by infusion of EBV-specific immune T cells [2,3]. These lesions comprise EBV-transformed B cells, which are phenotypically similar to lymphoblastoid cell lines (LCL) that are easily established following experimental infection of resting B cells with EBV *in vitro*. The growth-transformed LCL generally sustain a non-permissive infection with expression of a limited number of so-called latent genes. The viral genes expressed in LCL encode six nuclear proteins (EBNA-1, EBNA-2, EBNA3A, EBNA-3B, EBNA-3, EBNA-LP), four membrane proteins (LMP1, LMP2A, LMP2B, and BHRF1) and various non-coding RNAs [4]. These transformation-associated genes act co-operatively on the cell to enhance cell survival and proliferation (reviewed in [4,5]). In this fundamental respect, EBV differs from human α-herpesviruses (e.g. herpes simplex, HSV) and β-herpesviruses (e.g. human cytomegalovirus, HCMV) in amplifying the viral load in the infected host by replicating itself via cell proliferation-associated duplication of the viral genome, in addition to the lytic cycle virus replication displayed by all herpesviruses.

## 2 EBV latency *in vivo*

In normal persistent infection of the healthy host, the EBV genomes in the resting memory B cell compartment are mostly transcriptionally silent (reviewed, [4,6]). The potential for these EBV genomes to be reactivated to express the growth-transformation-associated genes poses the risk of oncogenesis. Indeed, EBV is associated with several tumours of B cell origin, and also other cell types, including: post-transplant lymphoproliferative disease, Burkitt's lymphoma, Hodgkin's lymphoma, T/NK cell lymphomas, nasopharyngeal carcinoma, and gastric carcinoma (reviewed [5]). One strategy employed by the virus to limit the extent and consequences of EBV-induced cell proliferation in the immunocompetent host, is to link growth-transformation to immunogenicity. Thus, the LMP1 viral oncoprotein, which displays signalling functions similar to a constitutively active receptor of the CD40/TNFR family, has oncogenic potential by enhancing cell survival through upregulation of anti-apoptotic cellular proteins, but it also upregulates components of the antigen processing pathways to enhance presentation of viral target epitopes to CD4^+^ and CD8^+^ immune T cells [7]. In addition, the transformation-associated viral proteins (most notably the EBNA-3A, -3B, and-3C family of nuclear proteins) are themselves the source of several immunodominant target epitopes for the T cell immune responses (reviewed, [8]).

In immunocompetent and asymptomatically infected hosts, direct evidence of EBV-infected B cells expressing a growth-transformed phenotype is rarely found. Such cells are, however, observed in tonsils of IM patients where ineffective immune responses allow limited outgrowth of EBV-transformed lymphoblastoid cells [9]. Indirect evidence for expression of the growth-transforming genes in immunocompetent hosts is provided by the invariable presence of antibodies, CD4^+^ T cells, and CD8^+^ T cells with specificity for one or more of these proteins (reviewed, [5,8]). It is presumed, therefore, that growth-transformed B cells must be present in sufficient numbers to continuously stimulate and maintain the immune responses which, while not able to eliminate EBV completely from the host, are usually sufficient to prevent the outgrowth of potentially cancerous cells.

How is it that EBV is never eliminated? One possibility is that the original virus infection does not actually persist for life, but that the host is periodically re-infected by virus from other infected individuals. Arguing against this possibility is the observed persistence and dominance of a single virus isolate in many individuals screened for more than 15 years [10]. Current opinions on the mechanism of EBV persistence differ in certain details [5,11], but it is generally agreed that expansion of infected B cell numbers in immunocompetent hosts occurs in the secondary lymphoid tissues, and that some of the growth-transformed cells must revert to a more restricted form of latency, ultimately in memory B cells and before re-entering the periphery, in order to escape immune T cell responses. Consistent with this view, analysis of EBV-infected cells in the peripheral blood reveals that the virus is confined to the memory B cell population and that none of the transformation-associated proteins is expressed; the EBV genome is either silent or restricted to non-coding RNA transcripts (reviewed, [4–6]). These latently infected cells are, therefore, immunologically silent. Even in immunosuppressed individuals prone to lymphoproliferative disease, the elevated viral load in the blood is due to an increased number of immunologically silent, latently infected resting memory B cells rather than to the presence of growth-transformed or lytically infected cells [12].

## 3 EBV lytic replication *in vivo*

Whilst EBV differs from α- and β-herpesviruses in its ability to growth-transform cells in culture and in its causative role in cancers, in other respects it is a typical herpesvirus, particularly with regards to the generation of new infectious virus progeny though the lytic cycle. The major site of lytic EBV replication is in the oropharynx, where infectious virus is detected in the saliva.

It is not certain precisely where, or in what cells, does lytic replication normally occur. As is often the case, lessons from pathological states have been applied to construct hypotheses on the mechanisms of asymptomatic persistence. Superficially, EBV appears to be lymphotrophic *in vitro* as a result of the restricted expression of CD21, the cellular receptor for the gp350 viral envelope glycoprotein [5]. Nevertheless, the presence of EBV in oral hairy leukoplakia of AIDS patients and in nasopharyngeal carcinoma and gastric carcinoma clearly shows that infection of epithelial cells is possible *in vivo*. The question is, whether infection of epithelial cells is a normal part of EBV persistence, or whether it is a rare event associated with pathogenesis. In fact, it is now known that very efficient infection of epithelial cells can be achieved *in vitro* following binding of EBV to CD21 on B cells and transfer via conjugates formed between the virus-loaded B cells and epithelial cells, or by conjugates formed between lytically infected B cells and epithelial cells [13]. This is consistent with the view that infection of epithelial cells is a normal feature of asymptomatic persistence in the infected host, which might only rarely give rise to pathogenic lesions. In Hairy Leukoplakia, the lesions on the tongue or in the oral cavity were characterized by foci of extensive lytic virus production in differentiating epithelial cells [14]. This paralleled observations on other viruses, e.g. papilloma viruses, where latent infection in the basal epithelium progressed to lytic virus replication in the upper, more differentiated layers.

From this background, the idea was promulgated that EBV normally persists as a latent infection of B cells, but that lytic virus replication occurs in epithelial cells of the oropharynx [5]. The problem is that EBV lytic cycle in epithelial cells has only rarely been demonstrated in tissues from immunocompetent individuals. Furthermore, if persistence in the B cell compartment serves any purpose, the virus must be able to escape from the B cell. It is a reasonable presumption that at some point, latently infected B cells might be reactivated into lytic cycle. If this were to occur in tissues in the oropharynx, then the virus progeny might represent that virus which is detected in the saliva of healthy immunocompetent individuals. Alternatively, the virus produced in the B cells may infect epithelial cells as an amplification step for enhanced virus production. As with epithelial cells, however, convincing demonstration of lytically infected B cells in tissues from immunocompetent individuals is rare. The most reliable reported studies have been on tonsils, which are assumed to be the site of virus entry during primary infection. Nevertheless, only occasional lytically infected cells have been observed in tonsil tissues, most often in lymphoid cells and very rarely in epithelial cells [15,16]. It is possible that the majority of lytic EBV replication occurs not in the tonsil, but elsewhere in the oropharynx.

Whilst the nature of EBV latency *in vivo* is relatively well understood, there are significant gaps in the experimental evidence supporting the models of lytic EBV replication in the healthy immunocompetent host.

## 4 Immune responses to lytic EBV proteins

In excess of 80 EBV proteins are expressed during lytic virus replication [17]. Activation of lytic cycle from latency is initiated by expression of two immediate-early proteins encoded by the *BZLF1* and *BRLF1* genes. Their Zta and Rta protein products are transcription factors which activate a cascade of viral gene transcription, starting with several early genes after about 2 h, followed by delayed early and late genes from about 4 h onwards. Maximum expression of late proteins, including structural viral capsid antigens (VCA) and envelope glycoproteins, is achieved within about 12 h after Zta and Rta expression [17,18]. Although virus release can be detected within 24 h, cells in lytic cycle can actually survive several days *in vitro* before dying [19]. Consequently, there is considerable opportunity *in vivo* for mounting effective immune responses to lytic cycle proteins to limit infectious virus production. As with other herpesvirus infections, the host mounts vigorous humoral and cellular immune responses to many lytic cycle antigens. Serum IgG antibodies to VCA are reliable indicators of the EBV carrier status of healthy infected individuals. Serum IgA antibodies to VCA and/or early antigens can be diagnostic for nasopharyngeal carcinoma; this is often interpreted as reflecting the mucosal origin of this EBV-associated tumour [5]. However, the emerging tumours are usually VCA-negative, and the IgA response is therefore most likely stimulated by lytic cycle activation in non-tumour cells at mucosal sites in these patients. Serum IgA antibodies to lytic cycle antigens are also generated during primary infection IM patients, again indicating lytic virus production in mucosal sites [20].

T cell responses to lytic cycle antigens represent a dominant fraction of the EBV-specific T cells generated during primary EBV infection manifest as IM [8]. Whilst some of these lytic antigen responses disappear with resolution of the disease, responses to some lytic cycle antigens still represent a significant proportion of the EBV-specific T cell responses in the normal persistent carrier-state. Interestingly, however, the primary CD8^+^ responses to EBV lytic cycle antigens show a skewed immunodominance for the immediate-early and early antigens, with remarkably few responses detected to late lytic cycle antigens [21]. This is now thought to reflect the existence of, and temporal expression of viral immune-evasion genes. Three such genes (*BNLF2a, BGLF5, BILF1*) were recently demonstrated to directly interfere with the HLA-I antigen presentation pathway to modulate the CD8^+^ immune responses to EBV, and the molecular functions of these genes will be discussed in more detail in the next section.

The EBV *BCRF1* late gene encodes a vIL-10 cytokine that has also been shown to downregulate cell surface HLA-1 through transcriptional inhibition of TAP-1 expression, although whether this actually impacts on T cell recognition of endogenous antigen was not tested [22]. The vIL-10 homologue shares some of the properties of the human IL-10 cytokine, and its effects on the immune system are multiple and complex. Indeed, vIL-10 has also been reported to stimulate the reactivation of anti-EBV T cell responses [23]. The EBNA1 nuclear protein, which is expressed both in latent and lytic forms of infection [17], is also able to interfere with antigen presentation although the mode of action is conceptually distinct from BNLF2a, BGLF5, BILF1, and vIL-10 in that it only affects the processing of antigenic peptides from the EBNA1 protein itself. This unusual mechanisms is the result of a large repetitive region in the *EBNA1* gene encoding a Gly-Ala repeat domain comprising about one-third of the EBNA1 protein, which interferes with proteasomal degradation of the EBNA1 polypeptide [24].

Modulation of CD4+ immune responses has been less well studied. Nevertheless, downregulation of HLA class II (HLA-II) transcripts by the Zta immediate-early protein [25] and the BGLF5 early viral protein [26], together with the steric interference with T cell receptor (TCR) recognition by physical association of the late viral BZLF2 protein (gp42) with HLA-II protein complexes [27,28], indicate that EBV has likewise evolved mechanisms to interfere with the HLA-II antigen presentation pathway.

There is also evidence that EBV lytic cycle gene products can evade anti-viral interferon responses. For example, the immediate-early Zta protein inhibits IFNγ-receptor expression by the infected cell [29]. This might directly impact on T cell recognition of lytic cells because one of the functions of interferons is to upregulate expression of HLA-I, HLA-II, adhesion molecules, and other components of the antigen presentation pathways.

## 5 Modulation of the HLA-I antigen presentation pathway by EBV lytic cycle proteins

**Fig. 1:**
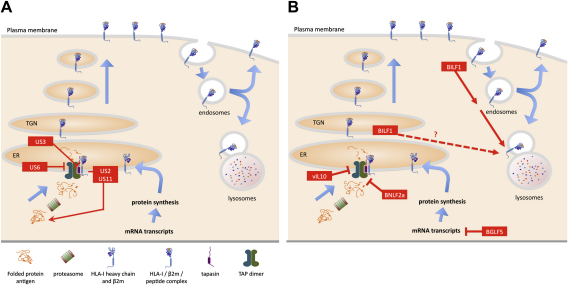
Schematic comparing the modulation of HLA-I antigen presentation by (A) HCMV and (B) EBV gene products.

The HCMV and murine CMV β-herpesviruses have become paradigms for viral interference with the HLA class-I (HLA-I) antigen presentation pathway (reviewed [30,31]). HCMV, for example, encodes at least 4 proteins that interfere with HLA-I antigen presentation at multiple points in the pathway from proteasomal degradation of target proteins through to peptide transport to the endoplasmic reticulum, formation of peptide/HLA-I/β_2_-microglobulin trimeric complexes in the endoplasmic, and transport to the cell surface for presentation to the TCR of CD8^+^ T cells (see Fig. 1A). The HCMV early protein, US6, inhibits peptide transport to the endoplasmic reticulum via the peptide transporter (TAP-1/TAP-2) complex, and early or late proteins affect HLA-I maturation either by retaining these molecules in the ER (US3), or inducing their retrograde transport to cytosol for proteasomal degradation (US2, US11).

Many other examples of interference with the HLA-I presentation pathway by viruses other than CMV have also been identified (reviewed, [31]). The search for equivalent mechanisms by EBV was initiated following the demonstration that EBV-infected B cells in lytic cycle showed downregulation of cell surface HLA class I expression that was at least in part due to impaired TAP-1/TAP-2 peptide transport function and to a hitherto unknown host protein-synthesis shutoff function [19,32,33]. Subsequently, the identification of at least three EBV proteins that limit the visibility of lytically infected cells to CD8^+^ T cells suggested that EBV has similar strategies to CMV for interfering with HLA-I antigen presentation, although they differ in the detail of individual molecular mechanisms (Fig. 1B). The current knowledge of the molecular functions of the BNLF2a, BGLF5 and BILF1 proteins will now be summarized:

## 6 BNLF2a

The immune-evasion function of BNLF2a was first identified through a targeted strategy based on the reasoning that such functions were most likely to map to genes unique to the γ_1_-herpesvirus genus [34]. Interestingly, of the 11 EBV genes unique to γ_1_-herpesviruses of Old World primates, at least 4 are now known to have immune-modulatory functions: the *BARF1* gene encodes a secreted protein able to bind Colony-Stimulating Growth Factor-1 and thereby inhibit macrophage activation [35]; the gp42 product of the *BZLF2* gene associates with MHC-II molecules to inhibit recognition by the TCR of EBV-specific CD4^+^ T cells [27,28]; *BILF1* encodes a vGPCR that targets HLA-I molecules for degradation (see below); and *BNLF2a* encodes a small, 60 amino acid residue membrane protein that localizes to the endoplasmic reticulum and physically associates with the TAP complex [34,36].

BNLF2a effects downregulation of cell surface HLA-I molecules and impairment of antigen presentation to CD8^+^ effectors by inhibiting peptide transport to the ER lumen via TAP [34]. Normal translocation of antigenic peptides from the cytosol to the ER via the TAP complex is initiated by the binding of peptides to cytosolic domains of TAP, followed by the binding and hydrolysis of ATP which promotes the opening of a transmembrane pore through which the peptides are then pumped. BNLF2a acts by binding TAP and inhibiting both peptide and ATP binding to TAP, thus efficiently blocking peptide transport [34,36]. BNLF2a therefore adds to a growing list of herpesvirus proteins that inhibit TAP function, although its mechanism of action differs from those previous identified. For example, the US6 protein of HCMV binds to an ER luminal loop of TAP and signals across the ER membrane to inhibit ATP binding to the cytosolic domain of TAP, thus inhibiting ATP hydrolysis but not affecting the ability of peptides to bind TAP [37]. Conversely, the ICP47 proteins of HSV-1 and -2 efficiently compete for peptide binding to TAP without affecting ATP binding [38,39], while the UL49.5 gene product of Bovine herpesvirus-1 affects neither peptide nor ATP binding to TAP, but still abrogates the peptide transport function and ultimately targets both TAP-1 and TAP-2 for degradation [40,41].

The binding of BNLF2a to TAP appears to stabilize expression of the viral protein since its expression levels in model cell lines devoid of TAP are substantially reduced, although the ER membrane localization of the residual BNLF2a remains unchanged [36]. Remarkably, however, during EBV lytic cycle in TAP-expressing B cells the levels of BNLF2a protein peak at around 6 h after induction of lytic cycle, thereafter declining rapidly to barely detectable levels by 12 h despite an only modest decrease in BNLF2a transcripts in this time-frame [18]. The mechanisms underlying this transient expression of BNLF2a protein during lytic cycle are unknown, but the implications for the immune-evasion strategy of EBV have been investigated. Thus, CD8+ T cell recognition of immediate-early and early EBV proteins in lytically infected B cells carrying a BNLF2a-deleted recombinant EBV was enhanced relative to recognition of those targets in lytically infected B cells carrying a wild-type EBV genome; in contrast deletion of BNLF2a did not enhance recognition of late lytic cycle antigen targets [18]. This fits with the transient nature of the BNLF2a protein expression, which declines as late lytic cycle proteins are expressed, and indicates that additional immune-evasion genes come into play as lytic cycle progresses. *BGLF5* and *BILF1* are two such genes.

## 7 BGLF5, a dual-function protein

The product of the *BGLF5* gene was originally identified as a DNase enzyme [42], which is homologous to the alkaline exonucleases that are highly conserved in all herpesviruses. These DNases are early lytic cycle genes, and they are critically required for efficient processing of the viral genome, as was originally shown for HSV-1 where *nuc* mutations in the *UL12* DNase gene resulted in a 10^−2^ to 10^−3^ reduction in infectious virus titres following lytic replication [43,44]. The *BGLF5* gene is likewise essential for efficient infectious virus production, being required for optimal viral DNA synthesis, genome encapsidation, and nuclear egress of the virus [45].

*In vitro*, herpesvirus DNases accept both double-stranded and single-stranded DNA as substrates, and they function optimally at alkaline pH in the presence of Mg^++^ divalent ions. They preferentially show 5′–3′ exonuclease activity, although weaker 3′–5′ exonuclease and endonuclease activities have also been observed. Sequence alignment of DNase proteins from several different herpesviruses first indicated 7 conserved motifs [46] and subsequent molecular modeling and phylogenetic analyses suggested that herpesvirus DNases belong to a restriction endonuclease superfamily characterized by a catalytic core motif, PD-(D/E)XK [47]. Site-directed mutagenesis of these defining residues in BGLF5 confirmed their importance for the alkaline DNase activity [48] and, more recently, the crystal structure of BGLF5 was determined to 3 Å resolution to confirm and greatly extend the details of these structure–function observations [49].

The alkaline DNase function of the EBV gene is therefore well established. However, a second less well understood function of this molecule was recently identified: a host protein-synthesis shutoff function which operates at the level of mRNA transcripts [26]. The identification of this second function for BGLF5 followed an earlier observation from the Ganem group that the DNase encoded by the *ORF37* gene of KSHV had dual host-shutoff and exonuclease functions; hence, the ORF37 protein was designated SOX [50]. In both BGLF5 [51] and SOX [52] proteins the exonuclease and host-shutoff functions are genetically separable. Furthermore, both BGLF5 and SOX were found to decrease expression of HLA-I molecules at the cell surface and to reduce antigen presentation to CD8^+^ effector T cells. This immune-evasion function mapped to the host-shutoff activity rather than to the exonuclease activity [51].

An additional shutoff function appears to be a feature of the DNases of the γ-herpesvirus genus [53]. In α-herpesviruses a host-shutoff function is mediated by a separate protein, typified by the *UL41* vhs protein of HSV-1, which is expressed as a late lytic cycle antigen and induces host-shutoff via an intrinsic RNase activity. Interestingly, the HSV-1 vhs protein has been shown to reduce expression of HLA-I, HLA-II, and several pro-inflammatory cytokines (reviewed in [54]). While BGLF5 and SOX similarly downregulate HLA-II as well as HLA-I [26,51], the extent of their immune-modulatory effects beyond impairing CD8+ T cell recognition has not yet been studied. The β-herpesviruses, do not encode any shutoff function, suggesting distinct evolutionary developments of DNase and host-shutoff functions in the three main herpesviruses families. Indeed, extensive phylogenetic analysis indicates that α- and β-herpesvirus DNases are more closely related to λ-bacteriophage DNase, while the DNases of γ-herpesviruses with acquired of host-shutoff function represent a more recent evolutionary divergence [47].

What is the mechanistic basis of the host-shutoff function of γ-herpesviruses DNases? Initially, it was thought that neither EBV-BGLF5 nor KSHV SOX had intrinsic RNase activity, indicating that their effect on global mRNA levels was mediated via modulation of cellular mRNA turnover pathways. With regards to the γ_2_-herpesviruses, KSHV and murine herpesvirus 68, the host-shutoff function of their SOX proteins appears to involve hyperadenylation of the poly-A tails of nascent cellular RNAs, promotion of their retention in the nucleus, together with a relocalization of a cytoplasmic poly-A binding protein to the nucleus, leading to instability of cytoplasmic mRNAs [53,55]. Furthermore, the recently resolved crystal structure of KSHV SOX shows that point-mutations specifically affecting the shutoff function mostly map away from the DNase catalytic core and are clustered at the surface of the protein, away from the catalytic core [56]. Dahlroth and colleagues have speculated that this surface region might be a point of interaction with cellular proteins or RNA.

**Fig. 2:**
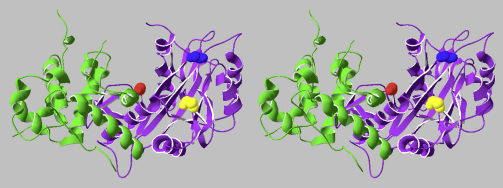
Residues in BGLF5 that, when mutated, affect both DNase and host-shutoff function (D203S; red), or selectively affect DNase (S280L; blue) or shutoff functions (K231M; yellow). The purple shaded regions of the BGLF5 cartoon structure represent the DNase catalytic core. The green shaded regions are the non-catalytic core domains mainly comprising the N-terminal domains, where in mutations selective for shutoff function are clustered in the KSHV SOX protein [56]. The structure of the BGLF5 protein was taken from PDB ID: 2W45 [49] (For interpretation of the references to colour in this figure legend, the reader is referred to the web version of this article).

A recent study by Buisson and colleagues, in which they resolved the crystal structure of BGLF5, indicates an additional mechanism of host-shutoff. An intrinsic RNase activity was observed in the presence of Mn^++^ ions, rather than the Mg^++^ ions preferred for DNase activity, which degraded unstructured mRNA but not structured tRNA [49]. This RNase activity was due to a 5′–3′ exonuclease activity, and was abrogated by a D203S mutation in the essential catalytic core motif (D203 …. E225-X-K227) of DNase activity. The *in vivo* significance of this intrinsic RNase activity, which may be up to 2 orders of magnitude weaker than the DNase activity *in vitro*, remains to be determined, particularly since it is not known whether the intracellular concentration of Mn^++^ ions is sufficient for RNase activity. At the time of writing this review, it is not known whether the γ_2_-herpesvirus SOX proteins share this Mn^++^-dependent RNase activity. There are, however, some indications that BGLF5 might differ from the γ_2_-herpesvirus SOX proteins in the mechanistic details of host-shutoff function. Thus, our attempts to extrapolate from the mutational studies on KSHV SOX [52] to engineer a mutant BGLF5 that selectively lost either DNase or shutoff function were unsuccessful (Zuo & Rowe, unpublished). Random mutagenesis of BGLF5 resulted in the generation of 67 mutants in which both DNase and shutoff function were impaired, but only one mutant (S280L) that was selectively impaired for DNase activity and another (K231M) that was selectively impaired for shutoff function [51]. In contrast, random mutagenesis of the KSHV SOX protein gave rise to 6 mutants with selective loss of shutoff function, one with selective loss of DNase function and about 20 with double loss of function (Dr Britt Glaunsinger, personal communication, and [52]). Furthermore, whereas the SOX mutations that selectively impaired shutoff function mostly localized to an area of surface away from the catalytic core [56], the K231 residue of BGLF5 locates to the edge of the DNase catalytic canyon (Fig. 2). While these data do not rule out the possibility of additional protein–protein interactions in the N-terminus region of BGLF5, they do point to the possibility of γ_1_- and γ_2_-herpesviruses having evolved distinct mechanisms for the acquisition of host-shutoff functions.

## 8 BILF1, a viral G-protein-coupled receptor (vGPCR)

Following a systematic screen of EBV lytic genes, *BILF1* was identified as a gene able to downregulate cell surface HLA-I expression, but not HLA-II, and to impair CD8^+^ T cell recognition of endogenously-processed antigen [57]. *BILF1* encodes a seven transmembrane spanning protein with structural and functional characteristics of a constitutively signalling vGPCR/chemokine receptor [58,59].

All herpesviruses encode at least one vGPCR, and their role in viral lytic cycle is thought to be to reprogramme the host cell through multiple signaling pathways [60,61]. Several vGPCRs have the potential to modulate the immune responses. For example, the US28 vGPCR of HCMV [62] and the U51 vGPCR of HHV6 [63] can modify the chemokine environment of infected cells by binding and internalizing MCP-1 (monocyte chemoattractant protein) and RANTES (regulated on activation, normal T cell expressed and secreted). In addition, the UL28 vGPCR of HCMV can transcriptionally regulate expression of MCP-1 [64,65], and the U51 vGPCR of HHV6 can transcriptionally regulate expression of RANTES [63]. To date, however, BILF1 is the only human herpesvirus vGPCR shown to target the HLA-I antigen presentation pathway; a property shared by the closely related vGPCR of the CeHV15 γ_1_-herpesvirus of Rhesus Old World primates, with 80% amino acid sequence identity to EBV BILF1, but not by the most closely related human herpesvirus vGPCR encoded by the *ORF74* of KSHV, which has just 15% sequence identity with EBV BILF1 [57].

Interestingly, downregulation of cell surface HLA-I expression can be induced by activation of the cellular CXCR4 GPCR by its CXCL12 ligand [66]. Mechanistically, this process involves physical association of CXCR4 with the β_2_-microglobulin, ubiquitination of HLA-I heavy chain, and endocytosis followed by degradation [66]. Superficially, this process is similar to BILF1 vGPCR-induced degradation of HLA-I in so much as BILF1 binds to the HLA-I/β_2_-microglobulin complex, accelerates loss of HLA-I molecules from the cell surface, and markedly reduces the half-life of HLA-I molecules [57]. However, a signaling-defective mutant of BILF1 retains its ability to downregulate HLA-I expression, and there no is no evidence that BILF1 induces ubiquination of MHC class I heavy chains above the levels seen in control cells lacking BILF1, and neither is there evidence that BILF1 associates with HLA-I via interaction with β_2_-microglobulin [57]. Nevertheless, it is notable that when co-expressed in the same cell BILF1 and CXCR4 show almost complete co-localization, both being predominantly localized to the plasma membrane, in contrast to KSHV-ORF74 and HCMV-US28 vGPCRs which exhibit a predominantly intracellular localization [59].

Many other viral gene products, all of which are structurally unrelated to GPCRs, have been shown to target HLA-I molecules for degradation as strategies for impairing antigen presentation to CD8^+^ T cells. They either induce endocytosis and lysosomal degradation of cell surface MHC class I complexes through RING-finger motif (e.g. the KSHV K3 and K5 proteins) or target MHC-I complexes for degradation through dileucine motifs in their cytosolic C-terminus (e.g. HIV-I nef, murine CMV gp48, and the HHV6 U21 proteins). The BILF1 vGPCR has neither a RING-finger motif nor dilucine motifs. However, our recent results with BILF1 mutants are consistent with the presence of C-terminus signal on the vGPCR which sorts HLA-I molecules on endosomes for degradation rather than recycling (Zuo and Rowe, unpublished). The HIV nef protein was one of the first viral proteins reported to induce endocytosis and degradation of HLA-I molecules from the cell surface [67], although more recent studies suggest that nef might act predominantly by diverting HLA-I molecules to endosomes/lysosomes before they reach the cell surface [68–70]. Since BILF1 is predominantly located in the plasma membrane, and can promote endocytosis of HLA-I from the cell surface, the original hypothesis for the mechanism of action of BILF1 was that it promotes degradation of HLA-I via endocytosis and lysosomal degradation [57]. Whilst this conclusion remains valid, our most recent data from mutant BILF1 proteins indicate that the mechanism of action of EBV vGPCR is more complex than was originally thought. Consistent with the fact that BILF1 can associate with immature HLA-I molecules in the ER shortly after synthesis [57], an additional mechanism of action might also involve diversion of HLA-I molecules for degradation before reaching the cell surface (Zuo and Rowe, unpublished).

## 9 Conclusions

That EBV has multiple proteins acting in concert on the HLA-I antigen pathway is significant in many respects. Firstly, it emphasizes that even when a herpesvirus has potent growth-transforming functions and the potential to cause cancer, the need for immune-modulating functions during its lytic replication stage is a paramount phenotype that unifies all herpesviruses. Secondly, it highlights the diversity of mechanisms that have evolved amongst herpesviruses to modulate the antigen presentation pathway. Despite related viruses targeting the same points in the antigen presenting pathway, the molecular properties of the viral proteins (which often have other functions besides immune-evasion), and the precise molecular mechanisms by which they act, suggest a bewildering complexity of independent evolutionary events driving the development of each herpesvirus. Even when a subgroup of viruses has acquired a second function in a viral protein with an already essential and conserved function in all herpesviruses, as is the case with the acquired shutoff function by DNases of γ-herpesviruses, it is possible that different molecular mechanisms evolved to effect this additional immune-evasion function. Finally, it should be noted that the multiple immune-evasion mechanisms of a virus do not necessarily afford absolute protection. The evasion mechanisms should be considered as strategies to dampen the efficiency of very potent immune T cell responses to lytic cycle gene products, rather than to completely evade them. This subtle modulation of immune responses plays an important role in enabling herpesviruses to persist as a largely asymptomatic infection.
